# PCNA promotes processive DNA end resection by Exo1

**DOI:** 10.1093/nar/gkt672

**Published:** 2013-08-10

**Authors:** Xiaoqing Chen, Sharad C. Paudyal, Re-I Chin, Zhongsheng You

**Affiliations:** Department of Cell Biology and Physiology, Washington University School of Medicine, 660 S. Euclid Avenue, St. Louis, MO 63110, USA

## Abstract

Exo1-mediated resection of DNA double-strand break ends generates 3′ single-stranded DNA overhangs required for homology-based DNA repair and activation of the ATR-dependent checkpoint. Despite its critical importance in inducing the overall DNA damage response, the mechanisms and regulation of the Exo1 resection pathway remain incompletely understood. Here, we identify the ring-shaped DNA clamp PCNA as a new factor in the Exo1 resection pathway. Using mammalian cells, *Xenopus* nuclear extracts and purified proteins, we show that after DNA damage, PCNA loads onto double-strand breaks and promotes Exo1 damage association through direct interaction with Exo1. By tethering Exo1 to the DNA substrate, PCNA confers processivity to Exo1 in resection. This role of PCNA in DNA resection is analogous to its function in DNA replication where PCNA serves as a processivity co-factor for DNA polymerases.

## INTRODUCTION

The cellular response to DNA double-strand breaks (DSBs) is crucial for maintaining genome stability, and its impairment causes a variety of systemic human diseases such as cancer, premature ageing, neurological and developmental disorders (1–4). DNA DSBs can be induced by ionizing radiation, some chemotherapeutic drugs, reactive oxygen species and collapse of replication forks. DSBs also occur naturally during meiosis and V(D)J recombination/Ig class switching during lymphocyte development (5,6). To combat the threat of DSBs, cells have evolved a highly sophisticated DNA damage response system that detects and repairs these breaks in an efficient and timely fashion (7,8). DSBs are repaired mainly by two competing pathways: non-homologous end joining and homologous recombination (HR). Non-homologous end joining occurs via direct rejoining of DNA ends, which sometimes requires limited DNA processing to generate end structures suitable for ligation. HR is initiated by a process called 5′-to-3′ DNA end resection that produces long 3′ single-stranded DNA (ssDNA) overhangs at a DSB end (9–12). This ssDNA structure is initially bound by RPA, and then replaced by Rad51, generating a Rad51-ssDNA filament. This filament serves as a key intermediate that searches for and invades into a homologous region, usually in the sister chromatid. Elongation of the 3′ end of the invaded ssDNA using the homologous sequence as a template and subsequent resolution of Holliday Junction structures and end ligation lead to the completion of HR (13,14).

DSB resection is also crucial for the activation of the checkpoint response, which coordinates DNA repair with other cellular processes and determines the fate of the inflicted cell (9–12,14). Checkpoint pathways are governed by the ATM and ATR protein kinases, which play redundant as well as distinct roles in regulating a range of cellular processes after DNA damage (15,16). Both ATM activation and ATR activation take place at the DNA damage sites; however, they require different DNA structures. Although ATM activation occurs on the dsDNA flanking the DSB ends and is coupled to Mre11-Rad50-NBS1 (MRN)-dependent damage sensing, ATR activation is believed to occur on RPA-coated ssDNA and is coupled to DNA end resection (17–20). By converting DSB ends into ssDNA structure, DNA resection also indirectly attenuates ATM activation (21,22). Thus, DNA end resection dictates both the pathway choice for DSB repair and the activation of different checkpoint pathways.

Recent studies in multiple organisms have led to the identification of a number of components such as MRN/MRX, CtIP/Sae2/Ctp1, Exo1, Dna2, BLM/Sgs1, RPA, LEDGF, SMARCAD1/Fun30 and SOSS1 in the DSB resection process (9,11,14). Studies in yeast indicate that DSB resection is initiated by MRX in cooperation with Sae2 followed by two parallel resection extension pathways that are mediated by Exo1 and Dna2, respectively (23–25). The Exo1 exonuclease plays a major role in the repair of DSBs and mismatches, and mutations in Exo1 or deregulation of its function are associated with increased cancer susceptibility, telomere defects and ageing (26). Although the nuclease activity of Exo1 is required for efficient DSB resection and repair, it is dispensable for mismatch repair (MMR) (27,28). This suggests that Exo1 plays distinct roles in different repair pathways. Consistent with this idea, Exo1 apparently interacts with a different set of protein partners in MMR and DSB repair (9,29,30). To date, the biochemical mechanisms and regulation of Exo1’s role in DSB resection and MMR remain incompletely understood. Exo1 exhibits limited processivity in DSB resection *in vitro* and has low abundance in cells, suggesting that there exist additional factors *in vivo* that promote its function in DNA end resection (31–33). Indeed, MRN/MRX, BLM, RPA and SOSS all exhibit stimulatory effects on Exo1-mediated resection *in vitro* by promoting Exo1 damage association and/or by enhancing Exo1 processivity (23,34–37). Recently, Rasmussen and colleagues have shown that a peptide containing a PCNA-interacting Protein Box (PIP-Box)-like sequence in the C-terminus of Exo1 can interact with the ring-shaped DNA clamp PCNA (38). Given the role of PCNA during DNA replication in promoting processive DNA synthesis by DNA polymerases, we hypothesize that PCNA may also function as a processivity factor for the Exo1 nuclease in DNA resection. Indeed, using human cells, *Xenopus* egg nuclear extracts and purified proteins, we have demonstrated that PCNA promotes Exo1’s function in resection by facilitating its damage association and increasing its processivity.

## MATERIALS AND METHODS

### Plasmids, siRNAs, shRNAs, antibodies and peptides

Human Exo1 cDNA, which was used to generate all the constructs encoding GFP-tagged, wild-type or mutant Exo1 in pEGFP-C1 through PCR or site-directed mutagenesis, was purchased from Invitrogen (Ultimate ORF IOH5832). To generate mCherry-PIPp21-WT-NLS and mCherry-PIPp21-MUT-NLS, DNA sequences encoding PIPp21-WT (DLSLSCTLVP-RSGEQAEGSPGGPGDSQGRKRRQTSMTDFYHSKRRAIAS) and PIPp21-MUT (DLSLSCTLVPRSGEQAEGSPGGPGDSQGRKRRATSATDAAHSKRRAIAS) derived from a modified p21 PIP-Box region (aa 111–160) were inserted downstream of the mCherry open reading frame in a pmCherry-C1-NLS vector. L157 and F159 in the C-terminus of the original p21 PIP-Box region were mutated to alanine to remove the CDK-binding site in the same region to avoid potential complications resulted from CDK binding (39). To generate Exo1(PIPFen1) constructs, the PIP-Box region of Exo1 (788QIKLNELWKNFGF800) was replaced by the wild-type (337QGRLDDFFKVTGS349) or mutant form (337AGRADDAAKVTGS349) of the PIP-Box sequence from Fen1 by overlapping PCR. To generate human Exo1 baculoviral expression vectors, PCR fragments encoding C-terminally His-tagged Exo1(WT), Exo1(ΔPIP) or Exo1(D173A) were inserted into the Gateway donor vector pDONR221 and then into pDEST8 through BP and LR recombinations, respectively, according to the manufacturer’s protocols (Invitrogen). GFP-PCNA in pEGFP-C1 was generated from a human PCNA cDNA. pET28a(+)-PCNA was used for generating recombinant His-PCNA in bacteria. FLAG-tagged human Dna2 expression construct was described previously and provided by Sheila Stewart (40). Control siRNAs (ON-TARGETplus Non-Targeting Pool, D-001810-10) and siRNAs targeting human PCNA (ON-TARGETplus SMARTpool, L-003289-00) were purchased from Thermo Scientific. Constructs encoding control shRNA (shLuc: GCAAGACATCCTACAAGAGAA) and shRNAs targeting Exo1 (shExo1-1: TGCAGACTGCTGCAAAGCTTT; shExo1-2: GCCGTGTTCAAAGAGCAATAT) in the LKO.1 vector were obtained from the Genome Institute at Washington University School of Medicine. Antibodies against human Exo1 (16253-1-AP) were purchased from ProteinTech Group. *Xenopus* Exo1 antibodies were raised in rabbits using a bacterially expressed His-tagged fusion protein containing the C-terminal fragment xExo1(400–734). *Xenopus* PCNA antibodies, which also recognize human PCNA, were provided by Johannes Walter and were also raised in-house in rabbits using His-xPCNA as antigen. xDna2 antibodies were provided by Hong Yan. xNBS1, xRPA, xKu70 antibodies have been described before (17,19,22). Anti-FLAG (F1804) and anti-MLH1 (M8445) antibodies were purchased from Sigma. Mouse monoclonal antibodies against γH2AX (07-164, Millipore) and PCNA (PC10, Santa Cruz Biotechnology) were used at 1:1000 and 1:50, respectively, for immunofluorescence staining. The PIPp21-WT (KRRQTSMTDFYHSKRRAIAS) and PIPp21-MUT (KRRATSATDAAHSKRRAIAS) peptides used as a competitor for PCNA-binding in *Xenopus* extracts were synthesized and purified (>90% purity) by Peptide 2.0 Inc.

### Cell culture, transfection, lentivirus production, laser irradiation, live-cell imaging and immunofluorescence staining

Human U2OS and HEK293T cells were cultured at 37°C with 5% CO_2_ in DMEM with 10% fetal bovine serum. Plasmid DNA was transfected into U2OS and HEK293T cells using Lipofectamine 2000 (Life Technologies) and TransIT-LT1 (Mirus) transfection reagents, respectively, according to the manufacturer’s instructions. Lentiviruses expressing shLuc (control), shExo1-1 or shExo1-2 in LKO.1 were generated in HEK293T cells by co-transfection of a lentiviral vector and packaging plasmids (pCMV-dR8.2 and pCMV-VSVG) using TransIT-LT1 transfection reagent, as previously described (22). Virus-containing supernatant was collected 48 and 72 h after transfection.

A customized laser microirradiation system consisting of an inverted microscope (Nikon), a laser ablation unit (Photonic Instruments) and microscope automation and imaging software (Metamorph, Molecular Devices) was described before (41). To introduce DNA damage to cells, a 551-nm dye laser was directed to irradiate cells cultured on 35-mm glass-bottomed dishes (MatTek Cultureware, P35G-15-14-C) in a line pattern. The laser energy delivered to each focused spot was set by an attenuator plate (50% transmission) and the number of pulses per spot (4). For cells transfected with fluorescently tagged Exo1 or PCNA, live-cell imaging was performed immediately after laser irradiation. The microscope stage is enclosed in a cell culture chamber environment (37°C, 5% CO_2_). Fluorescence images were captured every minute after laser irradiation except that the first images were taken 30 s immediately following laser irradiation. The damage association signal for GFP-Exo1 was measured in Metamorph by the fold increase in fluorescence intensity at the laser irradiation lines relative to undamaged areas. Each data point is the average of independent measurements of five cells.

To detect accumulation of endogenous Exo1 and PCNA at the DNA damage sites after laser irradiation, cells were fixed with 4% paraformaldehyde (Electron Microscopy Sciences) for 10 min and then permeabilized with phosphate buffered saline (PBS) containing 0.2% Triton X-100 for 10 min at room temperature. Subsequent immunofluorescence staining was performed as previously described (22). PCNA primary antibodies were used at 1:50. Mouse antibodies against γH2AX were used at 1:1000. xNBS1 antibodies, which also recognize human and mouse NBS1 proteins, were used at 1:1000 for immunofluorescence. Primary antibodies were detected with goat anti-mouse Alexa Fluor 488- and goat anti-rabbit Alexa Fluor 568-conjugated secondary antibodies (for Figure 2A) or with goat anti-rabbit Alexa Fluor 488- and goat anti-mouse Alexa Fluor 568-labeled secondary antibodies (for Supplementary Figure S4), used at 1:250 (Invitrogen). DNA was visualized with Hoechst 33342 (1 µg/ml) staining. Cells were then imaged using the Nikon microscope and MetaMorph software described earlier in the text.

### *Xenopus* egg extracts, immunodepletion, immunoprecipitation, immunoblotting and chromatin binding

*Xenopus* nuclear protein extract (NPE) was prepared from synthetic nuclei assembled in crude egg extract, as previously described (42). To deplete xExo1 from the *Xenopus* nuclear extract, 20 µl of protein A agarose beads coupled with 60 µl xExo1 antiserum were incubated with 100 µl of extract for 45 min at 4°C. Beads were then removed from the extract by low-speed centrifugation (5000 rpm) in a desktop microcentrifuge. The extract supernatant was then subjected to two additional rounds of depletion under the same conditions. To detect the interaction between PCNA and FLAG-tagged Exo1 and its mutants in human cells through co-immunoprecipitation, cultured HEK293T cells were washed once with ice-cold PBS and lysed in lysis buffer [20 mM Tris–HCl (pH 8.0), 1 mM EDTA, 150 mM NaCl, 0.5% NP-40, 1 mM PMSF] for 1 h. The cell lysate was centrifuged at 12 000 rpm for 15 min. The supernatant was pre-cleared with Protein A-Sepharose beads and then incubated with M2-conjugated Sepharose (Sigma) at 4°C for 2 h. For co-immunoprecipitation of endogenous PCNA and Exo1 in *Xenopus* extracts, Protein A-Sepharose beads pre-bound by anti-xExo1 or anti-xPCNA antibodies were incubated with the extract at 4°C for 2 h. Beads were then washed three times with lysis buffer, and the proteins bound were eluted with SDS sample buffer followed by SDS–PAGE. Immunoblotting was performed using DyLight 800- and DyLight 680-conjugated secondary antibodies (Pierce) and an Odyssey Infrared Imaging System (LI-COR Biosciences), as described previously (17,22). Chromatin-binding assays in the *Xenopus* extract were performed as described previously (19,22).

### Expression and purification of recombinant proteins and DNA substrate labeling

N-terminally His-tagged PCNA was expressed from pET28a(+) in *Escherichia coli* strain BL21(DE3). Recombinant, His-tagged Exo1(WT), Exo1(D173A) and Exo1(ΔPIP) were expressed in Sf9 insect cells using a Bac-to-Bac baculovirus expression system with Gateway Technology (Invitrogen), according to the manufacturer’s protocols. Briefly, C-terminally His-tagged Exo1 was cloned into the Gateway donor vector pDONR221 through PCR and BP recombination, and then transferred into pDEST8 through LR recombination. Subsequently, the destination vectors expressing Exo1 and its mutants were used to transform competent DH10Bac *E.coli* cells that contain a baculovirus shuttle vector and a helper plasmid to generate recombinant bacmid DNA. After verifying the inserts via a PCR-based approach, the bacmids were transfected into Sf9 cells using Cellfectin II transfection reagent (Invitrogen) to produce recombinant baculoviruses. After two rounds of amplification in Sf9 cells, the media containing baculoviruses were used to infect Sf9 cells for 72 h to produce recombinant proteins. Both His-PCNA and His-tagged, Exo1 and its mutants were affinity-purified using HisPur cobalt resin (Pierce), according to the manufacturer’s protocol. Purified proteins were dialyzed in PBS containing 10% glycerol and frozen in liquid nitrogen and stored at −80°C.

The 3′ ^32^P-labeled, 6 kb DNA substrate for resection was prepared by digesting an ∼6 kb plasmid pRS315 with XhoI followed by end filling using exonuclease-deficient Klenow Fragment in the presence of ^32^P-α-dCTP, dGTP, dTTP and ddATP (to prevent direct ligation of the DNA fragment in *Xenopus* extracts).

### DNA end resection assays in Xenopus extracts 

To assay DSB resection in *Xenopus* NPE, the 3′ ^32^P-labeled, 6 kb dsDNA fragment described earlier in the text was used as a DSB substrate. A typical resection assay contained 6 µl of treated or untreated NPE supplemented with an ATP regenerating system (2 mM ATP, 20 mM Phosphocreatine and 5 µg/ml Creatine Phosphokinase) and 1.5 µl (2 ng/ul) radiolabeled DNA substrate. To assess the role of PCNA-interaction in DNA end resection, NPE was pre-incubated with 200 µM PIPp21-WT or PIPp21-MUT peptide for 15 min before DNA substrate addition. To neutralize the effects of the PIPp21-WT, purified recombinant PCNA (60 µM) was added to NPE. Reactions were carried out at room temperature. After incubation, 1.5 µl of reactions were stopped at indicated times by incubating with 10 µl of stop buffer [8 mM EDTA, 0.13% phosphoric acid, 10% Ficoll, 0.2% bromophenol blue, 0.5% SDS, 80 mM Tris–HCl (pH 8.0)] supplemented with Proteinase K (2 mg/ml, to remove proteins) for 2 h at 37°C. Samples were then analyzed through 0.8% TAE-agrose gel electrophoresis overnight. The gels were then dried and exposed to autoradiography films.

*In vitro* resection reactions were performed using purified proteins, including His-tagged Exo1 proteins [Exo1(WT), Exo1(ΔPIP) or Exo1(D173A)], PCNA and the 3′ ^32^P-labeled 6 kb DNA fragment described earlier in the text. For a typical resection reaction, 15 nM Exo1-His, 300 nM His-PCNA monomer and 2 ng/µl DNA substrate were incubated in reaction buffer [20 mM Hepes (pH 7.5), 50 mM KCl, 0.5 mM DTT, 5 mM MgCl_2_, 5% glycerol] at room temperature. After incubation, reactions were stopped by adding stop buffer supplemented with Proteinase K at the indicated times and incubated at 37°C for at least 2 h. Samples were then analyzed on 0.8% TAE-agarose gels and autoradiography.

## RESULTS

### A C-terminal region of Exo1 containing a PIP-Box is required for its efficient damage association

**Figure 1.**
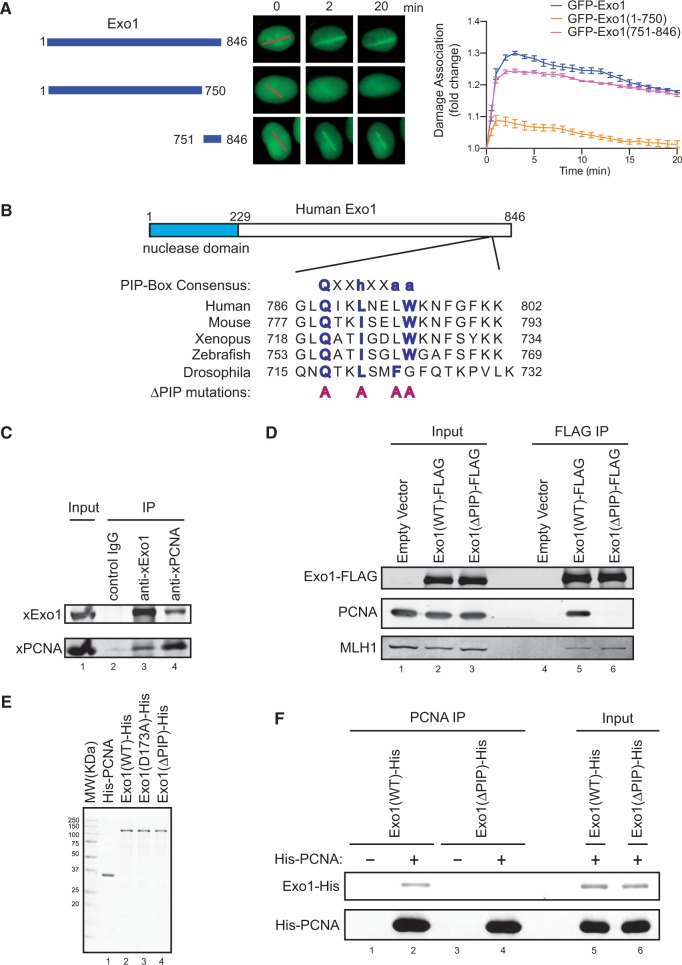
A C-terminal region of Exo1 containing a PIP-Box facilitates Exo1 damage association. (**A**). Left panel: Diagram of Exo1 and its truncation mutants. Central panel: Representative images for the damage association of GFP-Exo1 and its mutants shown in the left panel. Red lines indicate the sites of laser irradiation in cells. Right panel: Quantified results for the damage association of GFP-Exo1 and its mutants shown in the left panel during the first 20 min after laser irradiation. Each data point is the average of independent measurements of five cells. Error bars represent standard deviation. (**B**). A conserved PIP-Box-like sequence in the C-terminus of Exo1 in vertebrates and Drosophila. In the PIP-Box consensus sequence, ‘h’ represents a hydrophobic residue and ‘a’ represents an aromatic residue. In all the ΔPIP mutants described in this study, the four key residues are mutated into alanine. (**C**). Association of xExo1 with xPCNA in the *Xenopus* nuclear extract. (**D**). Association of PCNA with FLAG-Exo1, but not FLAG-Exo1(ΔPIP) without a functional PIP-Box, expressed in HEK293T cells. Mutation of the PIP-Box in Exo1 did not affect its interaction with MLH1. (**E**). Purified, recombinant His-tagged PCNA, Exo1(WT), Exo1(ΔPIP) and Exo1(D173A) proteins expressed in Sf9 cells. (**F**). Direct interaction between recombinant PCNA with recombinant wild type Exo1, but not the Exo1(ΔPIP) mutant lacking a functional PIP-Box.

To elucidate the Exo1-mediated resection pathway, we first examined the regulation of the damage recruitment of Exo1 in human cells. Both endogenous Exo1 and GFP-tagged Exo1 were efficiently recruited to DNA damage sites induced by laser irradiation (Supplementary Figure S1 and Figure 1A) (38,43,44). Interestingly, deletion of the C-terminal region (aa 751–846) of Exo1 drastically reduced the damage association of GFP-Exo1 (see result for GFP-Exo1(1–750) in Figure 1A). This C-terminal region fused to GFP was also efficiently recruited to DNA damage sites [see result for GFP-Exo1(751–846) in Figure 1A]. These data indicate that the C-terminal region of Exo1 facilitates its damage association.

Previously, a PIP-Box-like sequence (788QIKLNELW795) has been identified in this C-terminal region of Exo1 and the interaction between PCNA and Exo1 has been observed *in vitro* (38,45). This putative PIP-Box motif is conserved in vertebrates and Drosophila (Figure 1B). Thus, we hypothesized that PCNA interacts with Exo1 via this putative PIP-Box motif and that this interaction promotes Exo1 damage association. In support of this idea, we found that endogenous Exo1 was associated with PCNA in the NPE derived from *Xenopus* eggs (Figure 1C). In human cells, PCNA was also co-immunoprecipitated with FLAG-tagged Exo1 (Figure 1D). Importantly, mutation of four key residues in the PIP-Box sequence into alanine abolished the Exo1-PCNA interaction (but not the Exo1-MLH1 interaction) (Figure 1B and D). Furthermore, purified PCNA associated with recombinant wild type Exo1 protein, but not the PIP-Box mutant form of Exo1 *in vitro* (Figure 1E and F). Together these results demonstrate that PCNA directly interacts with Exo1 via the PIP-Box in the C-terminus of Exo1. In contrast to Exo1, no interaction between PCNA and Dna2 (another exonuclease that functions in parallel with Exo1 in DNA resection) was detected in human cells or *Xenopus* extracts (Supplementary Figure S2).

### PCNA-Exo1 interaction is required for efficient Exo1 damage association in human cells

**Figure 2.**
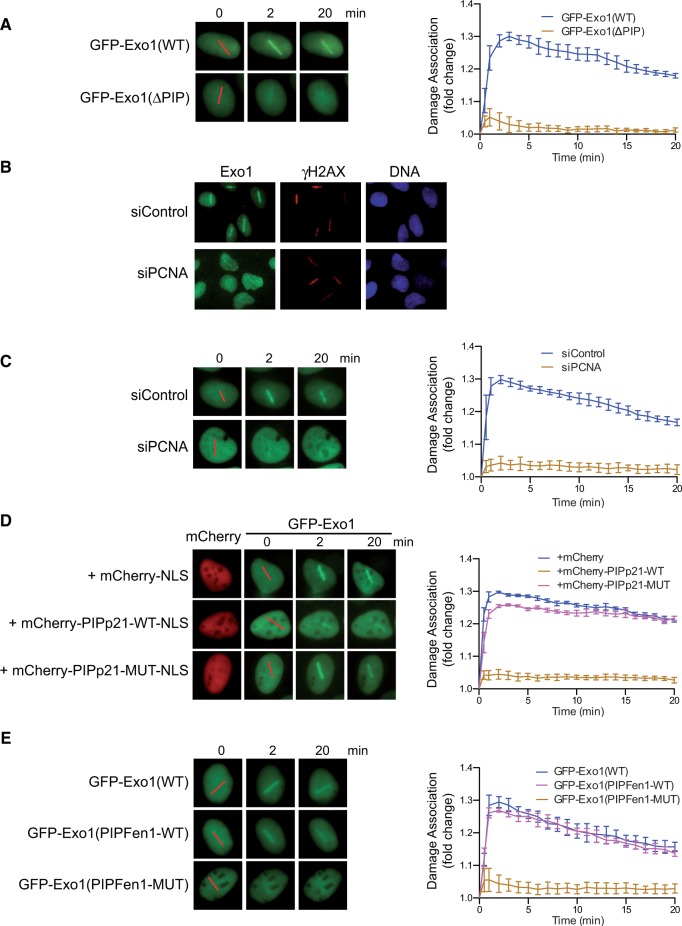
PCNA promotes the damage association of Exo1. (**A**)**.** Left panel: Representative images for the damage association of GFP-Exo1 and GFP-Exo1(ΔPIP) in human U2OS cells. Right panel: Quantified results for the damage association of GFP-Exo1 and GFP-Exo1(ΔPIP) during the first 20 min after laser irradiation. Each data point is the average of independent measurements of five cells. Error bars represent standard deviation. (**B**). Damage association of endogenous Exo1 in control- and PCNA-knowdown U2OS cells. γH2AX was used as a control to indicate the sites of DNA damage induced by laser irradiation. (**C**–**E**). Left panels: Representative images for the damage association of GFP-Exo1 in control-knockdown and PCNA-knockdown cells (**C**); of GFP-Exo1 in cells co-transfected with mCherry-NLS, mCherry-NLS-fused wild type or a mutant form of PIP-Box peptide derived from p21 (**D**); of GFP-Exo1, GFP-Exo1(PIPFen1-WT) and GFP-Exo1(PIPFen1-MUT) with the PIP-Box in Exo1 replaced with a wild-type or mutant PIP-Box in Fen1 (**E**). Red lines indicate the sites of laser irradiation in cells. Right panels: Quantified results for the damage association of the wild type or mutant GFP-Exo1 proteins depicted in the left panels during the first 20 min after laser irradiation. Each data point is the average of independent measurements of five cells. Error bars represent standard deviation. NLS, Nuclear Localization Signal.

To determine whether PCNA promotes the damage association of Exo1, we compared the damage recruitment of GFP-Exo1 and GFP-Exo1(ΔPIP) in transfected human U2OS cells. Disruption of the PIP-Box function in Exo1 severely reduced its damage association, suggesting that PCNA-interaction is important for Exo1 damage association (Figure 2A). Consistently, mutations in the PIP-Box also abolished the damage association of Exo1(751–846) (data not shown). Moreover, knockdown of PCNA using previously validated siRNAs also inhibited the damage association of endogenous Exo1 and GFP-Exo1 (Figure 2B and C and Supplementary Figure S3A) (46). Furthermore, we found that overexpression of an mCherry-NLS-fused PIP-Box motif derived from another PCNA-interacting protein p21 (mCherry-PIPp21-WT-NLS) in human cells blocked PCNA-Exo1 interaction and inhibited GFP-Exo1 damage association (Supplementary Figure S4 and Figure 2D). Importantly, this inhibition depends on the integrity of the competitor PIP-Box, as overexpression of a mutant form of the PIP-Box fusion protein (mCherry-PIPp21-MUT-NLS) or mCherry-NLS alone did not inhibit Exo1 damage association (Figure 2D). Lastly, a PIP-Box motif (wild-type, but not mutant) derived from another nuclease, Fen1, could functionally substitute the PIP-Box in Exo1 in supporting PCNA-interaction and Exo1 damage association (Supplementary Figure S5 and Figure 2E). Together, these data demonstrate that PCNA promotes the damage association of Exo1 in human cells.

### PCNA loads onto DSBs independently of Exo1

As Exo1 interacts with PCNA in the absence of DNA damage (Figure 1), we asked whether there is a reciprocal requirement for Exo1 in PCNA damage association. It has been previously shown that PCNA is also recruited to DNA damage sites in γ-irradiated human cells and fission yeast, suggesting that PCNA also loads onto DSBs (47,48). Consistent with this idea, both endogenous PCNA and GFP-PCNA were readily recruited to the laser-induced DNA damage sites in U2OS cells (Supplementary Figure S6A and B). PCNA also associated with sperm chromatin containing DSBs, but not intact chromatin, incubated with the nuclear extract derived from *Xenopus* eggs (Supplementary Figure S6C). Furthermore, PCNA bound to a bead-immobilized dsDNA fragment added to the *Xenopus* extract (Supplementary Figure S6D). These data indicate that like Exo1, PCNA also associates with DSBs after DNA damage.

**Figure 3.**
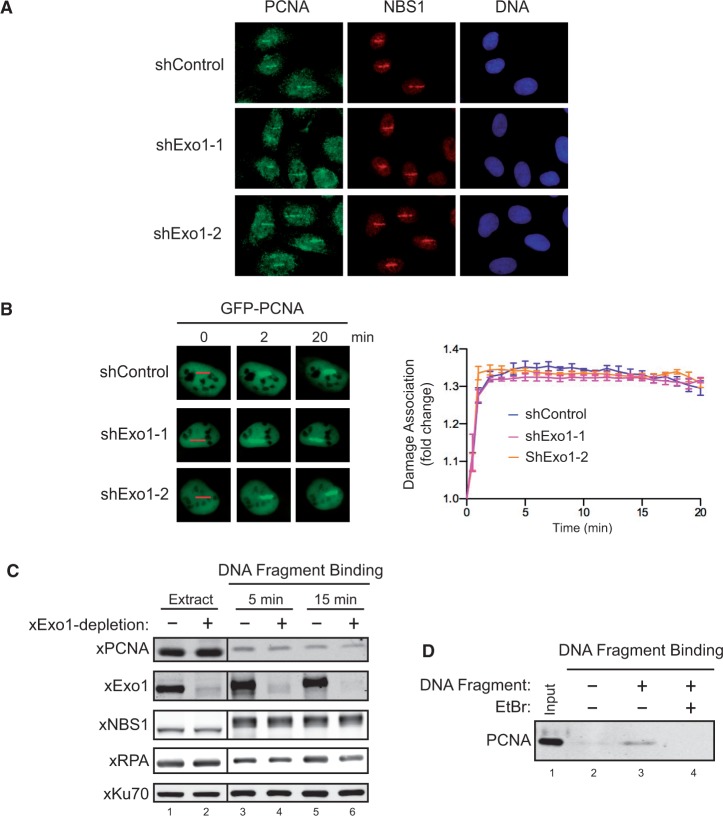
PCNA directly loads onto DSBs in an Exo1-independent manner. (**A**). Accumulation of endogenous PCNA at laser-induced DNA damage sites in control- or Exo1-knockdown U2OS cells. NBS1 was used as a control to indicate the sites of DSB damage in cells. (**B**). Left panel: Representative images for the damage association of GFP-PCNA in control- or Exo1-knockdown U2OS cells. Red lines indicate the sites of laser irradiation in cells. Right panel: Quantified results for the damage association of GFP-PCNA in control- or Exo1-knockdown U2OS cells during the first 20 min after laser irradiation**.** Each data point is the average of independent measurements of six cells. Error bars represent standard deviation. (**C**). Binding of xPCNA, xExo1, xNBS1, xRPA and xKu70 to a bead-immobilized 2 kb DNA fragment added to mock-depleted or xExo1-depleted *Xenopus* NPE. DNA-bound NBS1 was phosphorylated, resulting in its gel mobility shift. (**D**). Binding of purified His-PCNA to a bead-immobilized 2 kb DNA fragment *in vitro*, which was inhibited by Ethidium bromide (200 ng/µl).

To determine whether Exo1 is required for the loading of PCNA to DSBs, we examined the association of PCNA with DNA damage sites induced by laser irradiation in control- or Exo1-knockdown U2OS cells. Both endogenous PCNA and GFP-PCNA were efficiently recruited to DNA damage sites in Exo1-knockdown cells, and there was no significant difference in PCNA damage association in control- and Exo1-knockdown cells (Figure 3A and B and Supplementary Figure S3B). Moreover, depletion of Exo1 from the *Xenopus* extract also did not cause obvious effects on PCNA damage association (Figure 3C). Furthermore, purified recombinant PCNA protein alone could bind to a dsDNA fragment *in vitro* (Figure 3D). Taken together, these data indicate that PCNA loads onto DSBs after DNA damage and that this loading does not require Exo1.

### PCNA promotes Exo1-mediated DNA end resection

**Figure 4.**
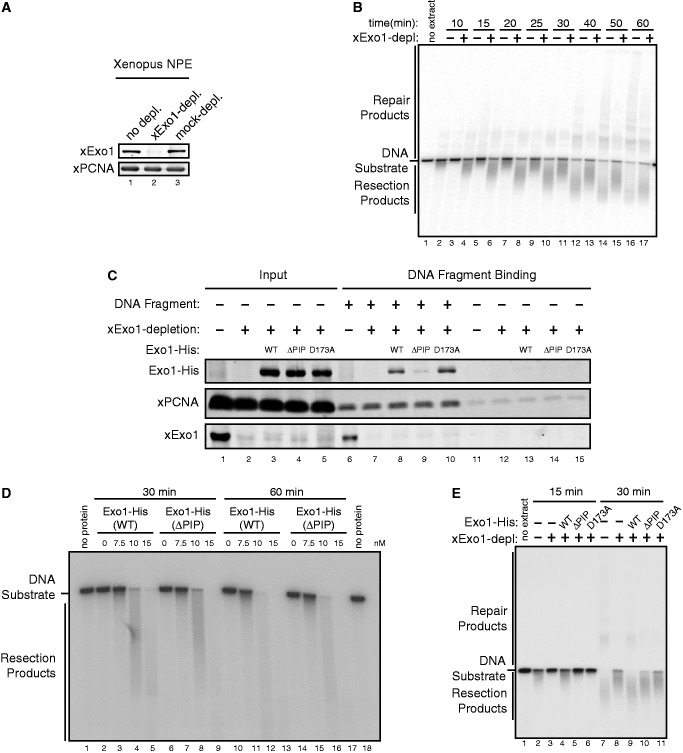
DNA end resection in the *Xenopus* nuclear extract by Exo1 requires its PIP-Box. (**A**) Immunodepletion of xExo1 from *Xenopus* NPE. (**B**) Depletion of xExo1 inhibited resection on a 3′ ^32^P-labeled 6 kb dsDNA fragment. Reactions were terminated at the indicated times, and resection products were resolved on a 0.8% agarose gel. (**C**) Recombinant human Exo1(WT) and Exo1(D173A) proteins, but not Exo1(ΔPIP), associated with a bead-immobilized DNA fragments in the *Xenopus* extract depleted of xExo1. (**D**) Exo1(WT) and Exo1(ΔPIP) exhibited similar levels of nuclease activity. Various amounts of purified recombinant Exo1(WT)-His or Exo1(ΔPIP)-His proteins were incubated with a 3′ ^32^P-labeled 6 kb dsDNA fragment. Reactions were terminated at the indicated times, and resection products were resolved on a 0.8% agarose gel. (**E**) Addition of recombinant human Exo1(WT), but not Exo1(ΔPIP) or Exo1(D173A), to xExo1-depleted extract rescued resection on a 3′ ^32^P-labeled 6 kb dsDNA fragment. Reactions were terminated at the indicated times, and resection products were resolved on a 0.8% agarose gel.

The role of PCNA in facilitating the damage association of Exo1 suggests that PCNA promotes DNA end resection. Directly testing this in cells could be difficult, as PCNA is also involved in DNA replication and is essential for cell viability, which could confound the functional analysis of PCNA specifically in DSB resection (49,50). To circumvent this issue, we chose to use the *Xenopus* nuclear extract, which maintains the proper DSB resection process but alone lacks DNA replication (42,51). To assay DNA end resection in the extract, we used a 3′-end ^32^P-labeled 6 kb dsDNA fragment and examined the length changes of the DNA substrate resulted from resection on agarose gels. Consistent with previous studies in *Xenopus* egg cytosol (52), we found that immunodepletion of xExo1 from the *Xenopus* nuclear extract partially inhibited DNA end resection (Figure 4A and B). The partial effects of Exo1-depletion on resection are consistent with the notion that Exo1 and Dna2 function in parallel in resection (23–25,51–55).

To assess the importance of PCNA-interaction in Exo1-mediated DNA end resection, we immunodepleted endogenous xExo1 from the *Xenopus* nuclear extract and then added recombinant, His-tagged Exo1(WT) or Exo1(ΔPIP) protein, and examined DNA end resection in these replacement extracts. Consistent with the above-described results in human cells, Exo1(ΔPIP) association with damaged DNA was severely impaired, compared with Exo1(WT) (Figures 1E and 4C). Importantly, Exo1(WT), but not Exo1(ΔPIP), could rescue DNA resection in xExo1-depleted extract, although Exo1(ΔPIP) has a similar level of nuclease activity as Exo1(WT) *in vitro* (Figure 4D and E). As a control, a nuclease-deficient mutant Exo1(D173A) added to the Exo1-depleted extract efficiently associated with damaged DNA, but it did not rescue DNA resection, as expected (Figure 4C and E). Together, these results strongly suggest that association with PCNA is required for Exo1 function in resection.

**Figure 5.**
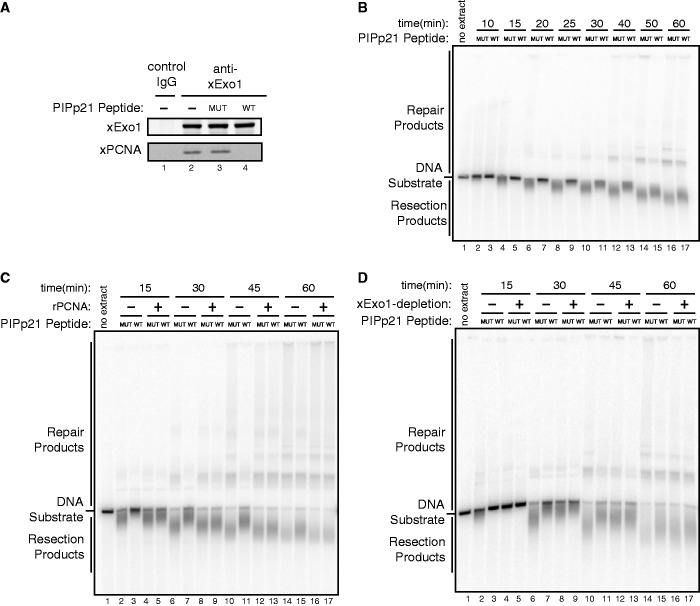
Disruption of Exo1-PCNA interaction inhibited Exo1-mediated DNA resection in the *Xenopus* nuclear extract. (**A**). Addition of a wild-type, but not a mutant form, of PIP-Box peptide derived from p21 inhibited the xExo1-xPCNA interaction in the extract. (**B**). Addition of a wild-type, but not a mutant form, of p21 PIP-Box peptide inhibited DNA resection in the extract. (**C**). Addition of excessive recombinant PCNA protein to the extract neutralized the inhibitory effects of the wild-type p21 PIP-Box peptide on DNA resection. (**D**). xExo1-depletion abrogated the inhibitory effects of the wild type p21 PIP-Box peptide on DNA resection in the extract.

As depletion of PCNA from the *Xenopus* nuclear extract was not successful [owing to its high concentration in the extract (∼ 15 µM)], we further tested the importance of PCNA-interaction in Exo1-mediated DNA resection using a synthetic peptide derived from the p21 PIP-box (PIPp21-WT) as a competitor to block Exo1-PCNA interaction. A mutant form of the peptide (PIPp21-MUT) that lacks the PCNA-binding capacity was used as a control. As shown in Figure 5A and B, the PIPp21-WT peptide, but not the PIPp21-MUT peptide, added to the extract abrogated the PCNA-Exo1 interaction, and partially inhibited DNA end resection. Importantly, addition of an excess amount of recombinant PCNA rescued resection in the extract supplemented with the PIPp21-WT peptide (Figure 5C). Immunodepletion of Exo1 from the extract completely abrogated the effects of the PIPp21-WT peptide on resection (Figure 5D). These results indicate that the inhibitory effects of the PIPp21-WT peptide on resection resulted from its inhibition of the Exo1-PCNA interaction. These data also strongly suggest that Exo1 is the major target of PCNA in resection [which is also consistent with the lack of interaction between PCNA and Dna2 (Supplementary Figure S2)]. Taken together, the results described earlier in the text further reinforce the idea that the PCNA-Exo1 interaction is crucial for Exo1-mediated DNA end resection.

### PCNA enhances the processivity of Exo1 in resection

**Figure 6.**
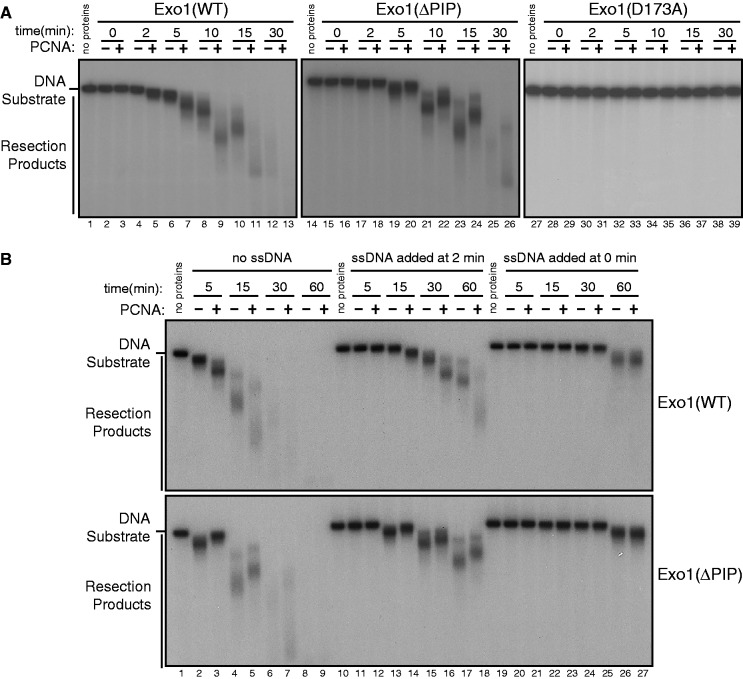
PCNA enhances the processivity of Exo1 in resection. (**A**). Resection of a 3′ ^32^P-labeled 6 kb dsDNA fragment by recombinant, His-tagged Exo1(WT), Exo1(ΔPIP) or Exo1(D173A) in the absence or in the presence of PCNA. PCNA: 300 nM (monomer); All Exo1 proteins: 15 nM. (**B**). In the *in vitro* resection reactions containing purified Exo1 and PCNA or containing Exo1 alone, circular ssDNA derived from pBluescript SK(−), which binds and sequesters Exo1, was added at 0 or 2 min. PCNA exhibited stimulatory effects on DNA resection by Exo1(WT), but not Exo1(ΔPIP), when the sequestering ssDNA was added at 2 min, but not at 0 min, presumably because Exo1 (WT) was tethered to the DNA substrate by PCNA during the first 2 min incubation. PCNA: 300 nM (monomer); All Exo1 proteins: 15 nM; pBluescript SK(−) ssDNA: 3 nM.

As PCNA is a ring-shaped clamp that can encircle dsDNA and can slide across the DNA, our results described earlier in the text suggest the possibility that PCNA promotes resection by tethering Exo1 to the DNA substrate, thereby increasing its processivity. To test this hypothesis more directly, we first reconstituted a DNA end resection reaction using purified, recombinant Exo1 and PCNA proteins and a 3′ ^32^P-labeled 6 kb dsDNA fragment as resection substrate. Consistent with our results in *Xenopus* extracts, purified PCNA drastically stimulated resection of the DNA fragment by Exo1(WT) (Figure 6A). In sharp contrast, PCNA did not stimulate, but actually inhibited, DNA resection by Exo1(ΔPIP), although this mutant has intact nuclease activity (Figure 4D and Figure 6A). The inhibitory effects of PCNA could be indirect; for instance, the occupation of the DNA fragment by PCNA may protect the DNA substrate from being attacked by Exo1(ΔPIP). The nuclease-deficient mutant Exo1(D173A) purified using the same procedure did not exhibit nuclease activity in the presence or in the absence of PCNA, demonstrating the specificity of the resection activities observed for Exo1(WT) and Exo1(ΔPIP) (Figures 1E and 6A). These results clearly indicate that PCNA stimulates Exo1-mediated DNA resection and that this stimulation is dependent on their direct interaction.

We next tested whether PCNA serves as a processivity factor for Exo1 in DNA resection, using a previously described procedure. In this procedure, Exo1 was first allowed to engage the radiolabeled dsDNA fragment in the presence of PCNA. Subsequently, an excess amount of unlabeled circular ssDNA, which binds and inhibits Exo1, was added to the reaction to sequester away any free Exo1 molecules in the reaction to prevent them from rebinding the dsDNA substrate (33,34). As shown in Figure 6B, when the sequestering ssDNA was preincubated with Exo1(WT) or with Exo1(ΔPIP) in the presence or in the absence of PCNA, resection of the radiolabeled dsDNA fragment was nearly completely inhibited during the first 30 min of incubation. Low levels of resection was detected 60 min after incubation; however, there was no difference between the reactions with or without PCNA (Figure 6B, Lanes 26 and 27). In contrast, when the sequestering ssDNA was added 2 min after the preincubation of Exo1(WT) with the dsDNA fragment and PCNA, the presence of PCNA significantly enhanced resection, although ssDNA still exhibited inhibitory effects on resection in the presence of PCNA (Figure 6B, upper panel). In contrast to Exo1(WT), PCNA did not stimulate, but slightly inhibited, DNA resection by Exo1(ΔPIP) under the same conditions (Figure 6B, lower panel). These results strongly suggest that PCNA promotes Exo1-mediated DNA resection, at least in part, by increasing the processivity of Exo1.

## DISCUSSION

**Figure 7.**
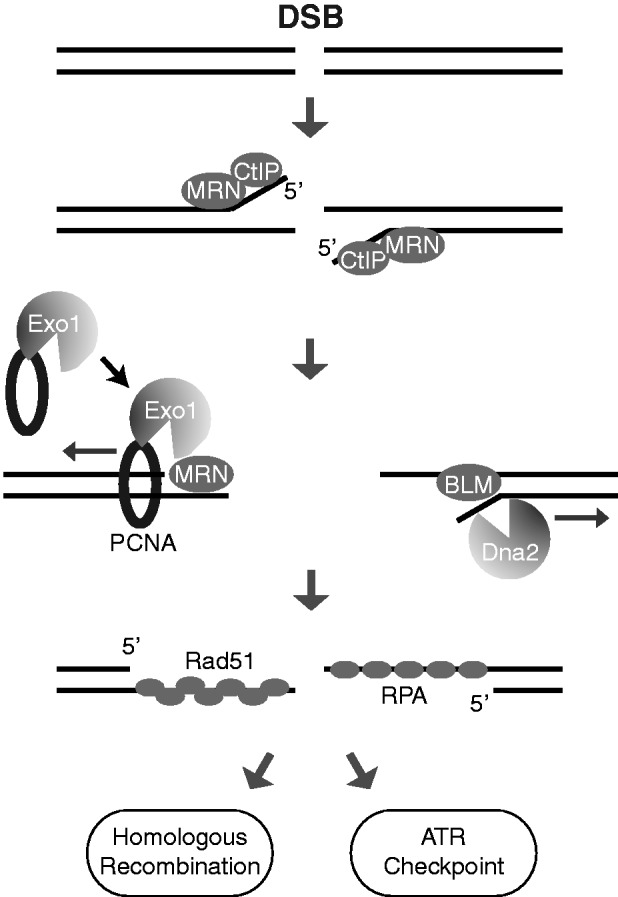
A model for DNA end resection. DNA end resection is initiated by MRN and CtIP through internal cleavage of 5′-strand DNA from the DNA ends. Subsequent resection extension is carried out by two parallel pathways mediated by Exo1 and Dna2, respectively. In the Exo1 pathway, PCNA loads onto the DSB from the ends and serves as a processivity factor for Exo1 in resection extension. MRN also enhances Exo1 processivity. The BLM helicase promotes Dna2-mediated resection extension. Binding of RPA to the 3′ ssDNA overhangs generated by end resection leads to recruitment and activation of ATR and subsequent checkpoint response. Replacement of RPA by Rad51 on the 3′ ssDNA overhangs leads to DNA repair by HR.

DNA end resection is a highly complex process that initiates homology-based DNA repair and activates the ATR-dependent checkpoint after DSB damage (10–12,14). Previously, we and Stephen Jackson’s group have shown that CtIP is a key factor in DNA end resection in metazoans (22,56). In this study, we have identified the ring-shaped DNA clamp PCNA as a new component in the Exo1-mediated resection pathway. Our results indicate that after DNA damage, PCNA loads onto DSBs and directly promotes the damage association of Exo1 and its function in DNA resection (Figure 7). In support of this idea, we have found that (i) Exo1 directly interacts with PCNA through a PIP-Box in the C-terminus of Exo1 (Figure 1); (ii) both Exo1 and PCNA are loaded onto DSBs (Figures 1A, 2 and 3); (iii) PCNA facilitates Exo1 damage association in cells, likely by promoting the retention of Exo1 at damage sites (Figures 2 and 4C); (iv) the PCNA-Exo1 interaction is required for Exo1-mediated resection in *Xenopus* extracts (Figures 4 and 5); (v) PCNA increases the processivity of Exo1 in DNA resection *in vitro* (Figure 6). Together, these data strongly suggest that PCNA serves as an intimate co-factor of Exo1 in DNA resection. Unlike Exo1, the Dna2-mediated DNA resection pathway apparently is not regulated by PCNA (Supplementary Figure S2 and Figure 5D).

A recent study by Rasmussen and colleagues suggests that the PCNA is not important for Exo1 damage association, as a PIP-Box mutant of Exo1 still exhibited damage association after laser irradiation (38). However, we found that knockdown of PCNA, mutation of the PIP-Box in Exo1 and overexpression of a competitor PIP-Box in human cells all inhibited Exo1 damage association (Figure 2). Furthermore, the PIP-Box in Exo1 is required for its efficient binding to damaged DNA in *Xenopus* extracts (Figure 4C). These data clearly demonstrate that PCNA promotes Exo1 damage association through direct interaction. The apparent discrepancy between our study and that of Rasmussen and colleagues may be in part caused by the differences in experimental conditions such as the laser power used for these two studies. For example, a high laser power would create a greater number of DSBs in the cell, which may result in a saturated GFP-Exo1 signal at DSBs and thereby mask the effects of PIP-Box mutation on Exo1 damage association. At the laser power used in this study, we observed only a low level of Exo1 damage association when the PCNA-Exo1 interaction was disrupted in cells (Figure 2). This observation is also consistent with the idea that PCNA serves as a processivity factor that retains Exo1 at DSBs after the initial damage recruitment of Exo1.

Although a role of PCNA in DSB resection has not been reported before, its function in DNA synthesis during DNA replication has been well documented. During DNA replication, PCNA is loaded onto the ssDNA–dsDNA junctions and functions as a processivity factor for DNA polymerases δ and ε. By tethering these polymerases to the DNA template, PCNA stimulates processive DNA synthesis (49,50). In addition to DNA replication, PCNA serves as a processivity clamp for DNA polymerase δ during the elongation of the invaded 3′ ssDNA end during HR (57). Our results described in this study strongly suggest that PCNA plays an analogous role in DNA resection: PCNA acts to increase the processivity of Exo1 in resection by tethering Exo1 to the DNA substrate (Figure 7). PCNA could directly bind to DSBs and increase Exo1 resection processivity in the absence of the clamp loader RFC, suggesting that RFC is not essential for PCNA function in DSB resection (Figures 3D and 6). In contrast, RFC is essential for PCNA loading and function in DNA replication. This is likely because PCNA can self-load onto DNA from the DSB ends in resection, while during DNA replication RFC is needed to open up the PCNA ring structure to load it onto the DNA template internally (58–60). However, we cannot rule out the possibility that RFC further enhances Exo1 resection *in vivo* by loading PCNA onto DSBs more efficiently from both DNA ends and internal regions flanking the DNA ends. Interestingly, like PCNA, purified human MRN can also increase the processivity of Exo1 in resection *in vitro* (34). The presence of these processivity factors, at least in part, explain the crucial contribution of Exo1 to DNA resection *in vivo*, despite its low abundance in cells and limited processivity *in vitro* (31,32). It is possible that PCNA may modulate Exo1 nuclease activity in cells in addition to its role in tethering Exo1 to the DNA substrate. A recent study on the N-terminal nuclease domain of Exo1 suggests the possibility that the C-terminus of Exo1 may serve as an autoinhibitory domain that binds (directly or indirectly) and suppresses the N-terminal nuclease activity of Exo1, and that the binding of MMR proteins such as the Msh2-Msh6 complex to the C-terminus of Exo1 relieves the inhibitory effects (61). Although this model awaits experimental validation, it is possible that the PCNA interaction with the C-terminus of Exo1 may stimulate Exo1 nuclease activity through a similar derepressing mechanism during DSB end resection; it will be interesting to test this possibility.

In the future, it will be interesting to determine whether the role of PCNA in Exo1-mediated resection is conserved in other organisms besides vertebrates. A PIP-Box-like motif can also be found in the C-terminus of Drosophila Exo1. Interestingly, a K/R-rich region immediately downstream of the PIP-Box in human Exo1 is present in both vertebrates and Drosophila (Figure 1B). This basic stretch is also present immediately downstream of the PIP-Box motifs of many other PCNA-interacting proteins such as p21, Cdt1 and Set8 (62). Although a canonical PIP-Box motif is not found in the C-terminus of *Caenorhabditis elegans* or yeast Exo1, PCNA may interact with Exo1 in these organisms through other regions or via an atypical PIP-Box in Exo1 (50). It will also be interesting to find out whether PCNA serves as a co-factor of Exo1 in other processes such as MMR, DNA replication and telomere regulation in which Exo1 is also implicated (26). In addition to PCNA and MRN, Exo1 function in DNA resection is regulated by other proteins, including CtIP, BLM, Ku, ATM, ATR, 14-3-3, SOSS1 and RPA (23,34–37,43,63–73). In yeast, Exo1 resection is also regulated by Rad53 (69–71). Although the functional relationships between these proteins in Exo1 resection remain to be elucidated, the multitude of regulation of the Exo1 pathway likely ensures efficient DNA end resection to promote DNA repair and checkpoint activation, and prevents unscheduled resection or over-resection that could cause deleterious consequences.

## SUPPLEMENTARY DATA

Supplementary data are available at NAR Online.

## FUNDING

National Institute of Health [R01GM098535]; American Cancer Society [IRG-58-010-52]; and a Siteman Career Award in Breast Cancer Research (to Z.Y.). The xExo1 antiserum was generated in Tony Hunter’s laboratory at the Salk Institute with support from National Institute of Health grant [R01CA80100]. Funding for open access charge: National Institute of Health.

*Conflict of interest statement*. None declared.

## Supplementary Material

Supplementary Data
