# Supplementary material for: PCNA promotes processive DNA end resection by Exo1
Source: Nucleic Acids Res. 2013 Aug 10;41(20):9325–38. doi: 10.1093/nar/gkt672 (PMC3814391; doi:10.1093/nar/gkt672)
Supplement: Supplementary Data [file supp_41_20_9325__index.html]

PCNA promotes processive DNA end resection by Exo1 — PCNA promotes processive DNA end resection by Exo1 — Supplementary Data 

# PCNA promotes processive DNA end resection by Exo1

## 

files

**Files in this Data Supplement:**

- Supplementary Data - pdf file
