# Supplementary material for: PCNA promotes processive DNA end resection by Exo1
Source: Nucleic Acids Res. 2013 Aug 10;41(20):9325–38. doi: 10.1093/nar/gkt672 (PMC3814391; doi:10.1093/nar/gkt672)

Supplemental Figure Legends

Fig. S1 (related to **Fig. 1). Association of Exo1 with DNA damage sites induced by laser irradiation in human cells.**

A). Accumulation of endogenous Exo1 with DNA damage sites at the indicated times after laser irradiation. γH2AX was used as a control to indicate the sites of DSB damage in cells.

B). Accumulation of GFP-Exo1 with DNA damage sites in a transfected cell at the indicated times after laser irradiation. The red line indicates the sites of laser irradiation in cells.

Fig. S2 (related to **Fig. 2**). **Dna2 is not associated with PCNA.**

A). Result of a co-immunoprecipitation experiment in *Xenopus* extracts indicates that in contrast to xExo1, which interacts with xPCNA, xDna2 is not associated with xPCNA.

B). Result of a co-immunoprecipitation experiment using FLAG antibodies indicates that PCNA associated with FLAG-Exo1, but not FLAG-Dna2, expressed in HEK293T cells.

Fig. S3 (related to **Fig. 2 and Fig. 3**). **Knockdown of PCNA or Exo1 in human cells.**

A). Knockdown of PCNA in U2OS cells using previously validated siRNAs from Thermo Scientific (ON-TARGETplus SMARTpool, L-003289-00). MLH1 was used as a gel loading control.

B). Knockdown of Exo1 in U2OS cells using shRNA-encoding lentiviruses. Due to its low abundance, endogenous Exo1 in control- or Exo1-knockdown cells was first enriched by immunoprecipitation followed by immunobloting. Chk1 in total cell lyates was used an input control.

Fig. S4 (related to **Fig. 2**). **Disruption of Exo1-PCNA interaction in human cells by overexpression of a competitor PIP box**

Result of co-immunoprecipitation experiment indicates that overexpression of mCherry-PIPp21-WT-NLS, but not mCherry-NLS or mCherry-PIPp21-MUT-NLS, inhibited the interaction between PCNA and GFP-Exo1 in U2OS cells.

Fig. S5 (related to **Fig. 2**). **The PIP-Box in Fen1 can functionally substitute the PIP-Box in Exo1 for PCNA-interaction.**

Co-immunoprecipitation of PCNA with GFP-Exo1(PIPFen1-WT), but GFP-Exo1(PIPFen1-MUT), in transfected U2OS cells.

Fig. S6 (related to **Fig. 3**). **Damage association of PCNA in human cells and *Xenopus* extracts**

A). Recruitment of endogenous PCNA to laser-induced DNA damage sites. γH2AX was used as a control to indicate the sites of DSB damage in cells.

B). Damage association of GFP-PCNA in a transfected U2OS cell at the indicated times after laser irradiation. The red line indicates the sites of laser irradiation in the cell.

C). Binding of xPCNA, xExo1, xNBS1, xRPA and xKu70 to chromatin treated with buffer or PflMI (to create DSBs) in *Xenopus* NPE.

D). Binding of xPCNA, xExo1, xNBS1, xRPA and xKu70 to a bead-immobilized 2 kb DNA fragment added to *Xenopus* NPE.

Chen et al., Fig. S1

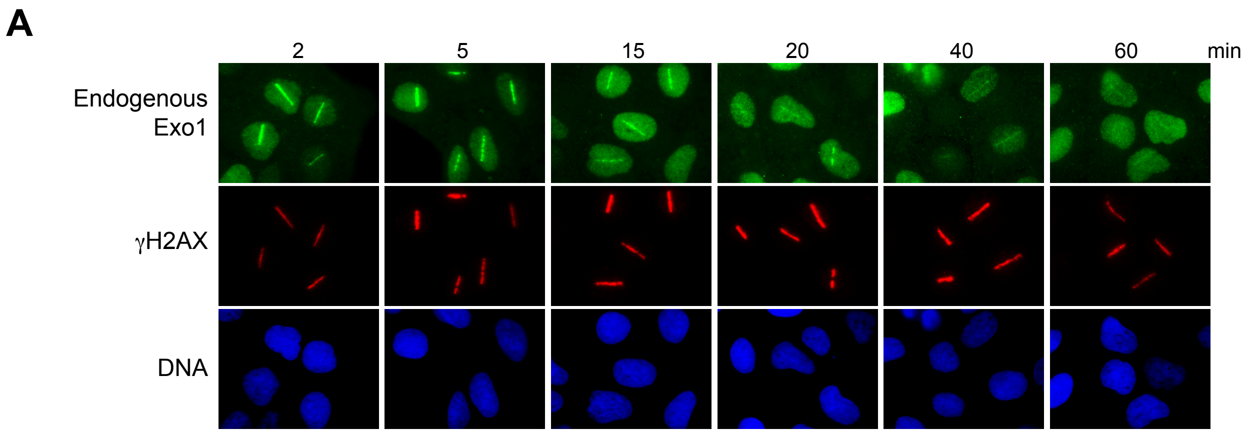

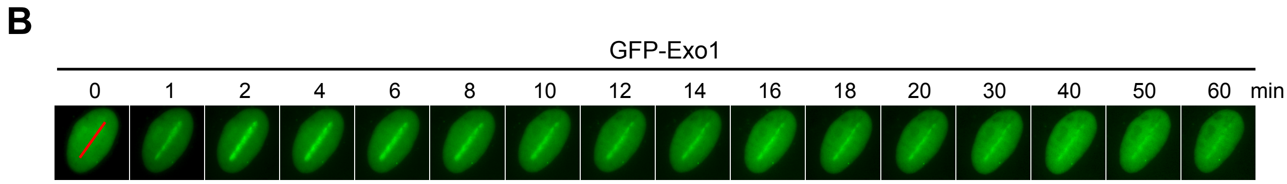

Chen et al., Fig. S2

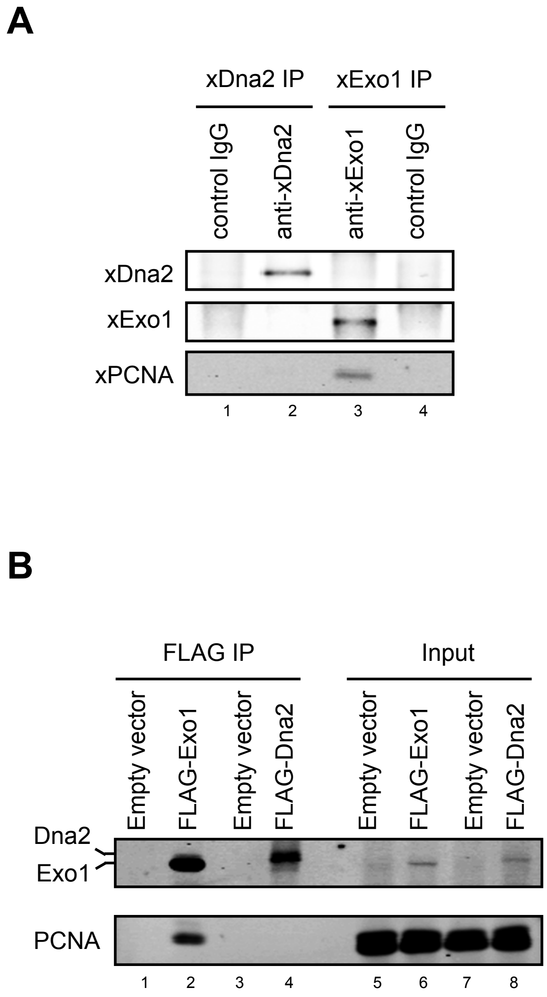

Chen et al., Fig. S3

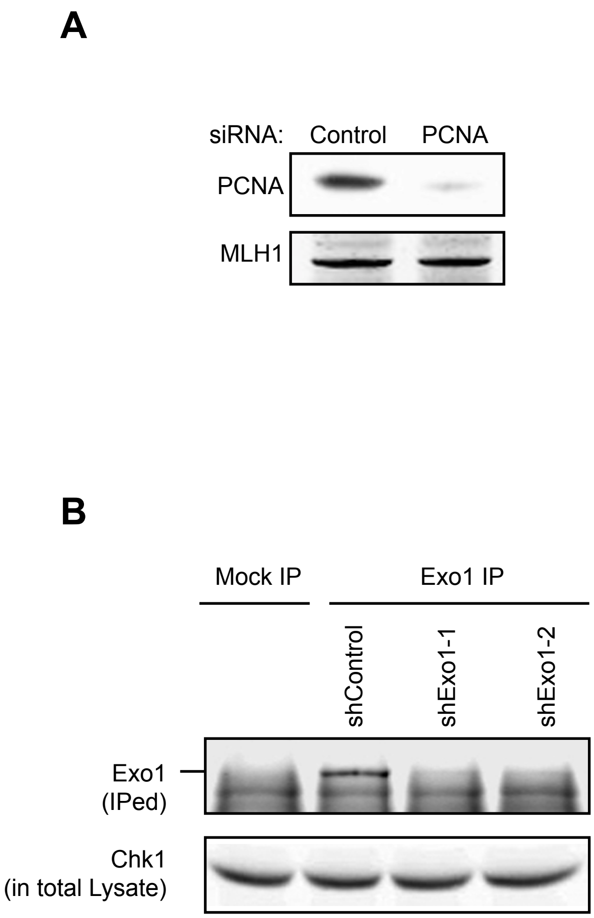

Chen et al., Fig. S4

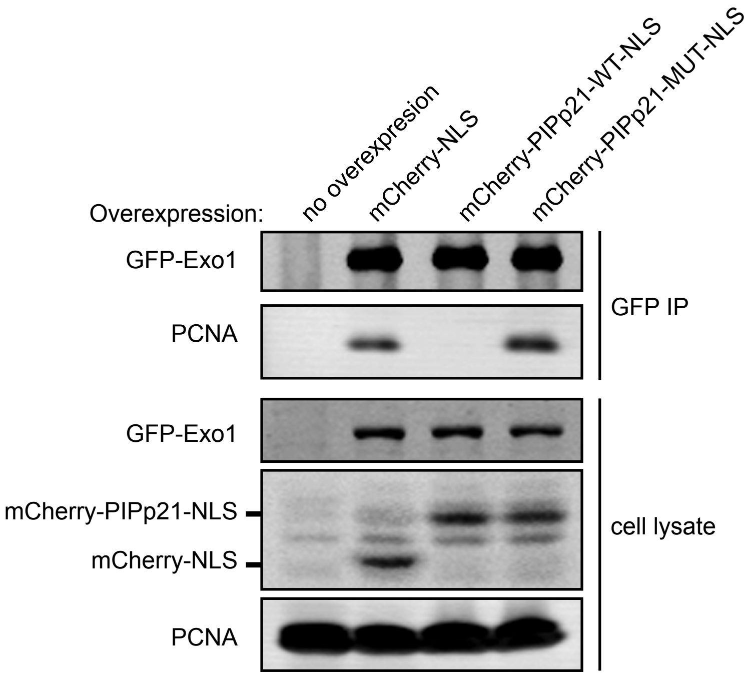

Chen et al., Fig. S5

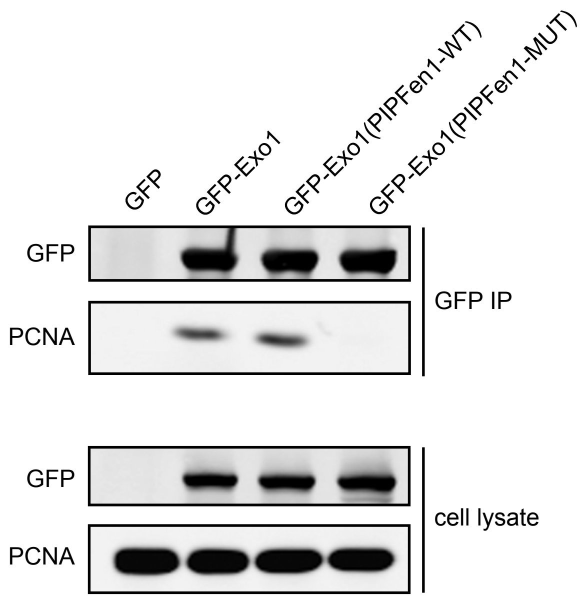

Chen et al., Fig. S6

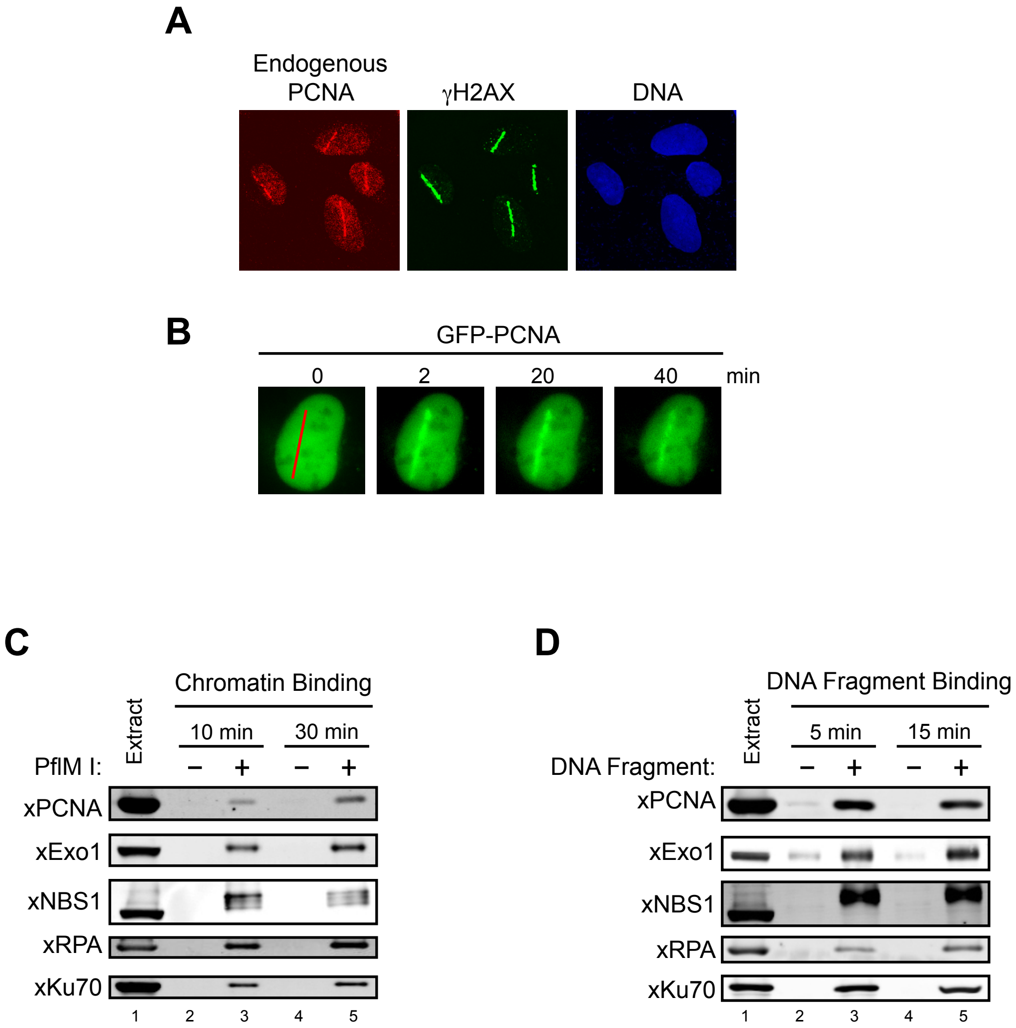

Supplement: Supplementary Data [file supp_gkt672_nar-01467-h-2013-File009.pdf]
